# A comparison of intervention and conservative treatment for angulated fractures of the distal forearm in children (AFIC): study protocol for a randomized controlled trial

**DOI:** 10.1186/s13063-015-0912-x

**Published:** 2015-09-30

**Authors:** Miriam Adrian, Daniel Wachtlin, Kai Kronfeld, Dirk Sommerfeldt, Lucas M. Wessel

**Affiliations:** Clinic for Pediatric Surgery, University Hospital Mannheim, Faculty of Heidelberg, Mannheim, Germany; Interdisciplinary Centre for Clinical Trials Mainz (IZKS Mainz), University Medical Centre, Johannes Gutenberg University, Mainz, Germany; Department of Pediatric Traumatology, Pediatric Clinic Altona, Hamburg, Germany

## Abstract

**Background:**

Angulated fractures of the distal forearm are very frequent lesions in childhood. Currently, there are no standard guidelines on whether these children should be treated conservatively with a cast; with reduction and a cast; or with reduction, pinning and a cast under anesthesia.

Minor prospective and retrospective studies have shown that the distal physis of the forearm possesses high remodeling capacity leading to reliable correction of malalignment.

The aim of this trial is to answer the question about whether operative and conservative treatment show equivocal results.

**Methods/Design:**

This is a prospective, multinational, multicenter, randomized, observer-blinded, actively controlled, parallel group trial, with 24 months of observation.

The primary objective of this trial is to assess whether or not the long-term functional outcome in remodeling patients is inferior to patients receiving closed reduction and K-wire pinning.

The trial should include 742 patients with acute fracture. The patients will be included in 30 medical centers in Germany, Switzerland and Austria.

All patients 5 to 11 years of age presenting at the emergency department with an angulated distal fracture of the forearm will be randomized online after informed consent.

The primary endpoint is the Cooney Score after 24 months. The secondary endpoint is the grade of radiological displacement at 12/24 months.

**Discussion:**

Therapy of angulated fractures is a matter of intensive debate. Primary manipulation and pinning under general anesthesia is recommended in order to avoid malalignment. No major study has proven the advantage of manipulation and pinning over immobilization alone. Should remodeling appear to be a safe alternative, manipulation under general anesthesia, K-wire pinning and removal of pins could be avoided, thus sparing significant costs.

**Trial registration:**

DRKS00004874, 30 October 2013.

## Background

Metaphyseal fractures of the distal forearm are the most frequent lesions in childhood and account for 20 to 25 % of all fractures [3, 6]. Half of these fractures are angulated, with the two bone segments remaining in contact.

Currently, there are no standard guidelines on how these patients should be treated. Treatment varies from simple immobilization to open reduction and plate osteosynthesis. Closed reduction of pediatric fractures commonly requires sedation and analgesia to achieve an anatomic reduction and to alleviate the child’s reaction to and recall of a painful and stressful situation. Inserted implants must be removed after bony healing and aneasthesia or sedation therefore is needed. Complications associated with procedural anesthesia include respiratory depression, hypoxia, hypotension, vomiting and aspiration [7, 16]. Therefore, some authors advocate nonmanipulative therapy for distal forearm fractures [8].

Primary closed reduction leads to secondary loss of reduction with the necessity of remanipulation under general anesthesia in >30 % of cases [15]. Although percutaneous K-wire pinning prevents redisplacement, effects on longer-term outcomes, including function have not been established [1, 13]. Inserted K-wired need to be removed.

Reliable remodeling of displaced radial fractures is often described, whereas the grade of possible remodeling differs in various publications [2, 10, 12, 14, 17]. In many uncontrolled or retrospective studies, although some children retained malalignment after an accident, after 2 years and until the age of 14, remodeling of the axis of up to 30° occurred due to growth.

Advantages of conservative therapy without manipulation are outpatient treatment, no need for anesthesia, cleaning of pins or wound control. Parents are often afraid of operations, and children, of manipulation while cleaning the pins. Disadvantages are the extended time of healing until remodeling is achieved and the visible angulation that can lead to questions and comments on incorrect treatment.

The advantage of the operation is the immediate transfer of the fracture into a stable anatomic position and less chance of secondary angulation. The disadvantage is the need of anesthesia for osteosynthesis, need for implant removal and inpatient treatment, as well as the cleaning of the pins or wound control to prevent infection.

### Primary objective

The primary objective of this trial is to compare whether patients from 5 to 11 years old with angulated fractures of the forearm present the same results in function and appearance after 2 years, no matter if they have been treated conservatively without reduction or operatively with reduction and K-wire osteosynthesis.

### Secondary objective

The secondary objectives of this trial are the assessment of safety and effectiveness and the satisfaction of patient and parents.

## Methods/Design

**Fig. 1:**
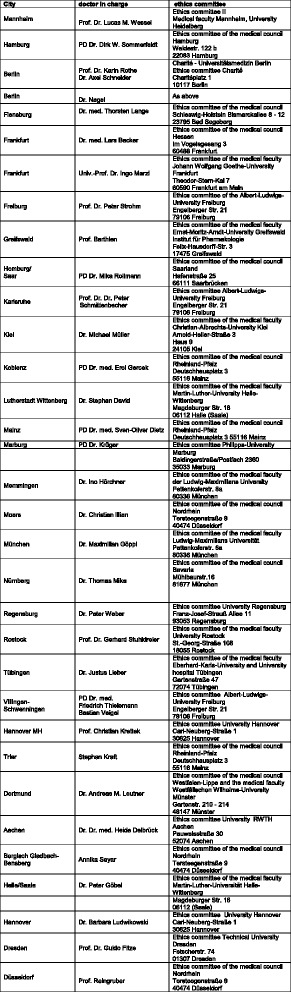
Ethical bodies

The protocol and all trial documents have been approved by the ethics committees responsible for the respective trial sites. The main vote was received from the ethical committee of the University Clinic Mannheim, faculty of the Ruperto Carola University of Heidelberg (code: 2013-544 N-MA). All ethical bodies that approved our trial are listed in Fig. 1.

### Inclusion criteria

Patients meeting all of the following criteria will be considered for admission to the trial:

1. Age 5 to 11 years
2. Distal metaphyseal fracture of radius or complete distal metaphyseal forearm fracture
  - 23-M/2-3 or 23-E/1-2 (according to AO classification)
  - Angulated radius or complete forearm fractures in the distal third of the bone
  - Angulated physiolysis with or without wedge of the metaphysis
3. Angulation up to 30°
  - Age 5 to 7 years 15°-30°
  - Age 8 to 11 years 10°-25°
4. Informed consent of child and parents

### Exclusion criteria

Patients presenting with one of the following criteria will not be included in the trial:

1. Torus fractures
2. Complete displaced fractures with shortening
3. Other osteosynthesis needed than K-wire
4. Neurologic disease
5. Metabolic bone disease
6. Neurovascular injuries
7. Multiple trauma

### Discontinuation criteria

A patient will be discontinued from the trial for any of the following reasons:

1. On request of the patient/parents
2. If the physician comes to the conclusion that continuing the trial is harmful to the patient’s well-being
3. If a treatment is needed that is not allowed in the protocol
4. Serious adverse events that are related to the trial
5. Safety reasons determined by the trial admission or the advisory board

### Treatment

Group 1 (experimental/conservative): Plaster immobilization without any reduction for 4 weeks, the kind of plaster (Paris, cast, forearm, upper arm or sandwich) is to be determined by the treating clinic. As no reduction is performed, no anesthesia is needed. After the fracture is immobilized with the plaster, the patient will leave the emergency department.

Group 2 (control): Closed reduction under anesthesia, percutaneous K-wire osteosynthesis with one or two wires (through physis or Kapandji), plaster (Paris, cast, forearm, upper arm or sandwich) is to be determined by the treating clinic. No other form of osteosynthesis is allowed. After the operation, the patient is treated on the ward for 1 or 2 days.

### Trial duration

The anticipated trial duration will be 60 months. Recruiting began in April 2014 and will be completed in March 2018. All patients will be monitored for 2 years.

### Number of patients

A total of 742 patients shall take part, with 371 per group. Recruiting is planned for 30 clinics.

### Primary endpoint

The primary endpoint is the Cooney Score after 24 months. If there is a visible malalignment, an X-Ray will be performed to confirm the exact degree of malalignement.

### Secondary endpoint

The secondary endpoints are as follows:

1. Completed Cooney Score after 3 and 12 months
2. Completed questionnaires CHC-SUN and ZUF-8 after 3, 12 and 24 months
3. Malalignment after 12 and 24 months
4. Second reduction
5. Need for reeapplied K-wire osteosynthesis
6. Growth disturbance
7. Complications (according to Dindo-Clavien 4)

### Randomization

After information is relayed by the physicians of the local clinic, the legal guardians and the child who wishes to participate must give oral consent. Online-based randomization is provided by the Interdisciplinary Center for Clinical Trials (IZKS), University Medical Centre of Mainz, and detailed information of the individual branch (operative or conservative) is given. All patients/caregivers, which are possible candidates for the trial, are informed about the aim of the trial, the possibility of conservative and operative treatment, the workflow and the randomization procedure. Written informed consent will be obtained from parents and patients before inclusion.

### Observer blinding

The person (usually a doctor) who determinates the Cooney score after 3, 12 and 24 months should not participate in the treatment of the patient and should not know which procedure was performed. In addition, available x-rays will be analyzed centrally to minimize bias.

### Trial schedule

**Fig. 2:**
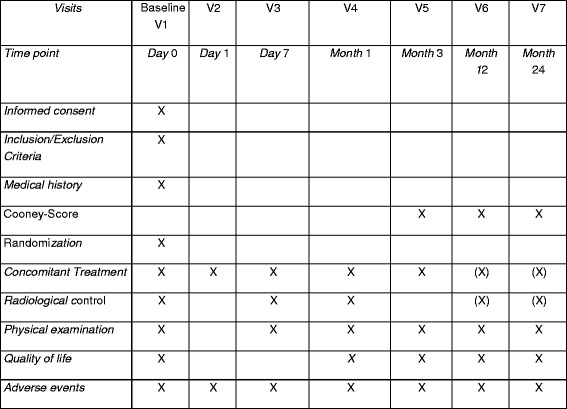
Work flow

For an overview of the schedule see Fig. 2.

Children between 5 and 11 years come to the emergency department with angulated fractures of the forearm. If all criteria are fulfilled and informed consent is given, the patient will be randomized.

After inclusion into the trial, dependent on the randomization result, patients are treated conservatively with a plaster (experimental group/group 1) or prepared for reduction and osteosynthesis under anesthesia (control group/group 2). A cast similar to the experimental group is applied.

For both groups, the kind of cast used (upper arm, forearm, sandwich, cast, or plaster of Paris) does not influence outcome of bony healing. In all controls, physical examinations in order to rule out complications of cast and serious adverse events are performed.

Patients included into the experimental group (1) go home after their treatment and come back the next day for clinical control. At this time-point quality-of-life questionnaires (ZUF-8 and CHC-SUN modified) are handed out. After 7 days, a clinical and X-ray control are performed in order to rule out secondary dislocation. If there is uneventful course of treatment, the next visit will be 4 weeks later for the next clinical and radiological control. If complete healing of the fracture is achieved, the cast is removed. Once again quality-of-life questionnaires are handed-out. Patients begin movements of daily routine, and physical exercise is postponed for another 2 to 4 weeks.

Patients included in the control group (2) stay on the ward after the operation for 1 or 2 days. Parents are instructed how to clean pin(s) or to perform wound controls. Exact reduction and position of K-wires is documented either during the procedure or on the next day. Quality-of-life questionnaires are handed out. At the next visit 4 weeks later radiological control and pin removal are performed, so far bony healing is documented. Patients begin with movements of daily routine, and physical exercise is postponed for another 2 to 4 weeks. The next quality-of-life questionnaires are handed out.

Three months after trauma, a next clinical control as well as assessment of the Cooney score and quality of life (CHC-SUN and ZUF-8) is conducted [5, 11]. 12 and 24 months after trauma similar visits in order to assess the same parameters are scheduled.

### Quality assurance

Clinical on-site monitoring in all trial centers is done by personal visits of clinical monitors according to the standard operating procedures of the IZKS. The monitor will check the informed consent forms and will review the entries into the case report form (CRF) on the basis of source documents. The physician allows the monitor access to all essential documents and provides support to the monitor. The IZKS Mainz assists the physician in conducting the study according to the protocol, as well as to meet regulatory and ethical requirements.

### Data management

Data management will be done by the IZKS. All participating clinics will enter their collected information in the CRF. Data will be exported into the statistical analysis system and checked for plausibility, consistency and completeness. Any missing data or inconsistencies will be reported back to the respective site and clarified by the responsible investigator. All collected data will be processed according to the German Data Protection Law and handled in strictest confidence.

### Power calculation/analysis

All patients presenting a Cooney Score of at least 90 after 24 months and, if clinically obvious, a radiologic displacement not exceeding 5°, will be compared between treatment groups by calculating a one-sided 97.5 % confidence interval of the difference of rates according to Farrington and Manning [9]. If this interval is located completely above the noninferiority bound of -5 %, noninferiority of the experimental intervention will be claimed. The primary analysis population is the per-protocol population, comprising all randomized patients without major protocol violations and at least one Cooney score radiological displacement measurement after intervention.

The primary endpoint is the validated Cooney-Score. This score considers subjective (pain, strength, activity before and after trauma) as well as objective (range of motion) parameters and has been validated in several trials that also included children and adolescents. As it is assumed that differences between the treatment groups with respect to the Cooney Scores fully disappear at (and not clearly before) 2 years after surgery/immobilization, an earlier time was not chosen for primary analysis.

Cooney Scores will be calculated according to the standard approach of aggregating the single items.

### Safety

Serious Adverse Events will be reported within 24 hours of the initial observation to the IZKS. An independent scientific advisory board will monitor and supervise the progress of the trial. Therapeutic complications will be documented and analyzed according to the Dindo-Clavien classification [4]. Tables and listing of adverse events will be provided.

## Discussion

Incomplete fractures and complete fractures without shortening of the distal forearm in children are very frequent lesions, and therapy is a matter of intensive debate.

Primary manipulation and pinning under general anesthesia is recommended in order to avoid malalignment. Nevertheless, many centers treat patients only with immobilization and achieve good results. Until now no randomized control was able to show advantage of manipulation and pinning over immobilization without reduction. In this trial, metaphyseal distal radius and forearm fractures angulated up to 30° will be treated in cast without reduction and compared to similar fractures treated by closed reduction, pinning and cast.

Should remodeling appear to be a safe alternative, manipulation under general anesthesia, K-wire pinning and removal of pins could be avoided, thus sparing significant costs.

## Trial status

At the time of submission, 30 trauma centers have been initiated and 42 patients included. Centers in Austria and Switzerland are preparing for initiation.

## Abbreviations

DFG: Deutsche Forschungsgemeinschaft (German funding committee)
DRKS: Deutsches Register klinischer Studien (German Clinical Trials Register)
K-wire: Kirschnerdraht
ZUF-8: Zufriedenheitsfragebogen (standarized questionnaire of satisfaction)
